# Anticancer Evaluation of *Adiantum venustum* Don

**DOI:** 10.4103/0975-1483.76419

**Published:** 2011

**Authors:** D Viral, P Shivanand, NP Jivani

**Affiliations:** *Department of Pharmaceutical Chemistry, Smt. R. B. P. Mahila Pharmacy College, Atkot, India*

**Keywords:** *Adiantum venustum*, Cancer, flavonoids, lipid peroxidation, terpenoids

## Abstract

Cancer is a malignant disease that is characterized by rapid and uncontrolled formation of abnormal cells which may mass together to form a growth or tumor, or proliferate throughout the body. Next to heart disease, cancer is a major killer of mankind. This study aims at a preliminary phytochemical screening and anticancer evaluation of *Adiantum venustum* Don against Ehrlich Ascites Carcinoma in animal model. The findings indicate that ethanolic extract of *A. venustum* Don possesses significant anticancer activity and also reduces elevated level of lipid peroxidation due to the presence of terpenoids and flavonoids. Thus, ethanolic extract of *A. venustum* Don could have vast therapeutic application against cancer.

## INTRODUCTION

The chemotherapy of neoplastic disease has become increasingly important in recent years. The relatively high toxicity of most anticancer drugs has fostered the development of supplementary drugs that may alleviate this toxic effect or stimulate the regrowth of depleted normal cells. Plants have a long history of use in the treatment of cancer, and they have played a vital role as a source of effective anticancer agent. It is significant that over 60% of currently used anticancer agents are derived, in one way or another, from natural sources, including plants, marine organism, and microorganisms.[1] It was also observed from Ayurvedic literature and ethanobotanical studies that the plant *Adiantum venustum* Don is very useful in treating tumor, prevention of hair from falling, and as a diuretic, but no scientific investigation has been carried out.[2–4] Therefore, it was thought worthwhile to carry out preliminary phytochemical screening and screening of *A. venustum* Don for anticancer activity against Ehrlich Ascites Carcinoma in animal model.

## MATERIALS AND METHODS

### Plant source

**Figure 1:**
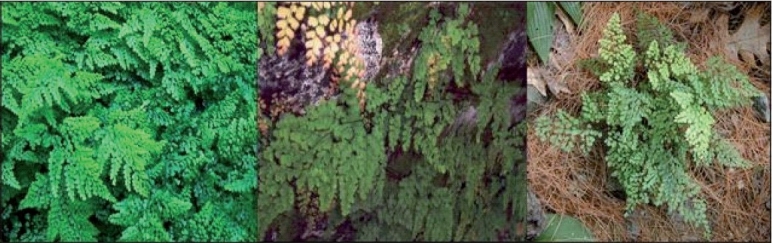
Plant images

The leaves and stem of *A. venustum* Don [Figure 1] were collected from Kolli Hills, Namakkal District, Tamilnadu, India, and were authenticated. Reference number of the authentication report is BSI/SC/5/23/05.06/Tech/603.

### Extraction procedure

The leaves and stem of *A. venustum* were dried under shade, mixed together, and then made in to a coarse powder in a mechanical grinder. The powder was passed through sieve no. 40 and stored in an airtight container for further use. The dried powder material (150 g) was defatted with petroleum ether (60–80°) to remove waxy substances and chlorophyll. The marc, after defatted with petroleum ether, was dried and extracted with ethanol (99.9% v/v) in a Soxhlet extractor for 72 h. The solvent was then distilled off, and the resulting semisolid mass was dried in a vacuum evaporator to get a yield of 14% w/w.[5,6]

### Phytochemical identification tests

Various chemical tests were performed for the identification of phytoconstituents in Pet. ether and ethanolic extract of the leaves and stem of *A. venustum* following the standard procedure.[7,8]

### Anticancer activity

#### Toxicity evaluation (LD_50_): (Karber’s methods)[9–11]

Swiss albino mice weighing 20–25 g were used for the study. Animals were fed a standard pellet and water and maintain at 24–28 °C temperature, 60–70% relative humidity and 12 h day and night cycle. Animals ascribed as fasted were deprived of food for 16 h, but had free access to water.

Fifty-four mice including both male and female weighing 20–25 g were selected for the study. Overnight faster mouse were divided into nine groups including four for PEETR and four for EETR and one control group each consists of six mice. Different doses of extract (200, 500, 1000, and 2000 mg/kg) were administered to nine experimental groups and control group received vehicle.

The animals observed continuously for their general behavior, such as motor activity, tremors, convulsions, straub reaction, Pilo – eraction, loss of lighting reflex, sedation, muscle relaxation, hypnosis, analgesia, ptosis, lacrimation, diarrhea, skin color, and mortality intermittently for next 24 h.

#### Animals

Male Swiss albino mice, weighing between 18 and 25 g, were used for this study. They were maintained under standard environmental conditions and were fed with standard pellet diet of water *ad libitum*. The mice were acclimatized to laboratory condition for 10 days before commencement of experiment. All procedure described were reviewed and approved by the Institutional Animal Ethical Committee of Smt R. B. P. Mahila Pharmacy College (rbpmpc/09/1025).

#### Cancer cell line

EAC cells were obtained from Amala Cancer Research Center, Kerala, India. They were maintained by weekly intraperitoneal inoculation of 10^6^ cells/mouse.

#### Preparation of extract drug and mode of administration

In the present anticancer study, ethanolic extract of *Adiantum venustum* (EEAV), in the dose of 150 and 250 mg/kg, were prepared as suspension by dispersing the ethanolic extract in a mixture of propylene glycol and sterile physiological saline containing Tween 20 (1:3) to get the desired concentration.[12,13]

#### Tumor transplantation

Ehrlich’s Ascites Carcinoma was maintained by serial transplantation from tumor-bearing Swiss Albino mice. Ascetic fluid was drawn out from tumor-bearing mice at the log phase (day 78 of tumor bearing) of the tumor cells. The tumor cell number was adjusted to 2 × 10^6^ tumor cells/mL. Sample showing more than 90% viability was used for transplantation. Each animal received 0.2 mL of tumor cell suspension containing 2 × 10^6^ cells/mL intraperitoneally.[11]

#### Drug treatment schedule

Male swiss albino mice were divided into five groups (*n* = 8). All the groups were injected with EAC cells (0.2 mL of 2 × 10^6^ cells/mouse) intraperitoneally except the normal group. This was taken as day 0. From the first day normal saline (0.9% NaCl), 5 mL/kg of body weight was administered to group 1 and propylene glycol 5 mL/kg was administered to group 2 (cancer control) for 14 days intraperitoneally. Similarly ethanolic extract of *A. venstrum* don at various doses (150 and 250 mg/kg/mouse/day) were administered to animals of groups 3 and 4, respectively. Standard drug vincrystine (0.8 mg/kg/day/mice) was administered to the group 5. After administration of last dose followed by 18 h fasting, four mice form each group were sacrificed for the study of anticancer activity, hematological, and liver biochemical parameters. The remaining animals in each of the groups were kept to check the mean survival lime (MST) and percent increase in life span of the tumor-bearing hosts.[12–14] Various parameters such as body weight of animals, life span of animals, cytological studies of cell lines, hematological parameters, RBC, WBC, hemoglobin, differential count, and biochemical parameters were evaluated in this study.

Anticancer effect of EEAV was assayed by observation of change with respect to body weight, ascitic tumor volume, packed cell volume, viable and nonviable tumor cell count, mean survived time (MST), and percentage increase in life span (% ILS).[12]

#### Tumor cell volume and packed cell volume

The mice were dissected to collect ascitic fluid from peritoneal cavity and centrifuged to determine packed cell volume at 1000 rpm for 5 min.[12] The transplantable murrain tumor was carefully collected to measure the tumor volume.

#### Viable and nonviable cell count

Viable and nonviable cell counting of the ascetic cell was done by staining with tryphan blue (0.4% in normal saline), dye exclusion test, and count was determined in a Neubauer counting chamber. The cells that did not take up the dye were viable and those that took the stain were not viable.[14]

#### Mean survival time and percent increased in life span

The effect of EEAV on tumor growth was observed by MST and % ILS. MST of each group continuing four mice were monitored by recording the mortality daily for 6 weeks and % ILS was calculated by using following equation.[15,16]

MST = (Day of first death + Day of last death)/2.

$$\%\;\mathrm{ILS}\;=\;\left\{\frac{MST\;of\;treated\;group}{MST\;of\;control\;group}\right\}1\;\times \;100.$$

#### Effect of EEAV on hematological parameters

Blood was collected from each mice by intracardial puncture with blood anticoagulant (heparin), white blood cells (WBCs), red blood cells (RBCs), hemoglobin, and differential count were determined[17] in group comprise of

- Tumor-bearing mice (control),
- tumor-bearing mice treated with EEAV (100 mg/kg/mice/day),
- tumor-bearing mice treated with EEAV (200 mg/kg/mice/day), and
- normal group.

#### Biochemical assay

After the collection of blood samples, the mice were killed, and their liver was excised. The isolated liver was rinsed in ice-cold normal saline followed by cold phosphate buffer having pH 7.4, blotted dry, and weighed. A 10% w/v homogenate of liver was prepared in ice-cold phosphate buffer (pH 7.4), and a portion were utilized for estimation of lipid. Other portion of the same, after precipitation of proteins with trichloro acetic acid (TCA), was used for the estimation of glutathione and the remaining homogenate was centrifuged at 1500 rpm at 4 °C for 15 min. The supernatant, thus obtained, was used for the estimation of superoxide dismutase, catalase, and protein content.[18]

### Statistical analysis

The experimental result were expressed as mean ± SEM. Data were assessed by the Student *t*-test, *P* < 0.05 was considered as statistically significant.

## RESULTS

Phytochemical screenings suggest that ethanolic extract of plant contain terpenoid, phytosterols, flavanoid, and saponin which are believed to be the main potential for anticancer activity [Table 1].[19,20]

**Table 1:** Result of phytoconstituent identification tests of ethanol extract of *Adiantum venustum* Don

Phytoconstituent	Phytosterol	Flavonoids	Triterpenoids	Saponin
Ethanol extract	+	+	+	+

+ represents present, – represents absent

### Anticancer activity

#### Toxicity evaluation (LD_50_)

**Figure 2:**
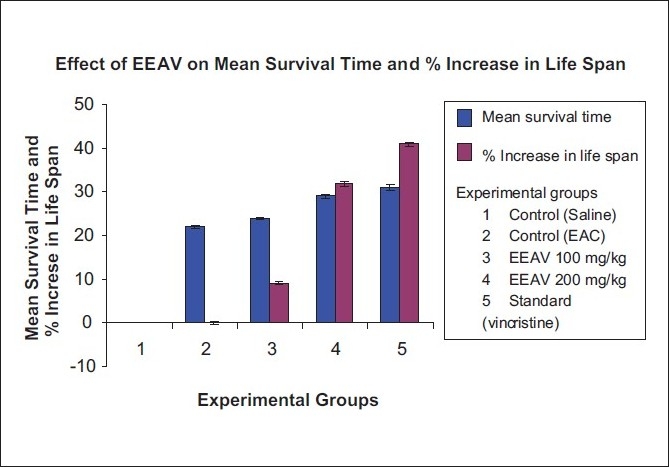
Graphical representation of Mean survival time and % increase in life span of mice

**Figure 3:**
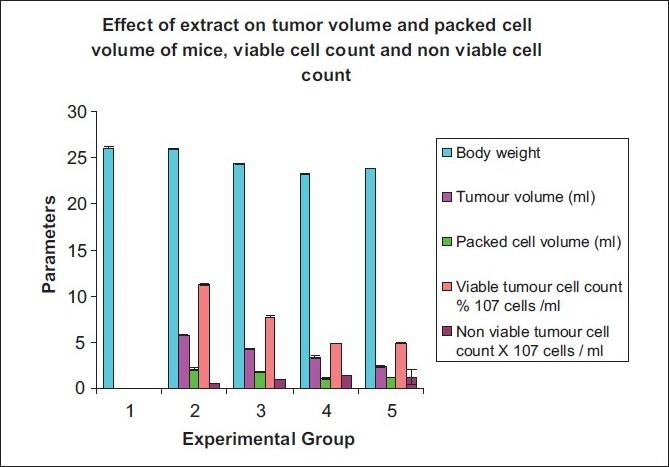
Graphical representation of effect of EEAV on tumor volume and packed cell volume of mice, viable cell count and non viable cell count

In acute toxicity study, the given extract of *A. venustum* did not show any mortality up to the dose of 2000 mg/kg. It is safe dose that was determined by organization for economic cooperation and development (OECD) guidelines. The extract shows sedation, hypnosis, and mild muscle relaxant property. Administration of EEAV reduces the tumor volume, packed cell volume, and viable tumor cell count in a dose-dependant manner when compared to EAC control mice. In EAC control mice, the median survival time was 22 ± 0.25 days. Whereas, it was significant increased median survival time (24 ± 0.33, 29 ± 0.49) with different doses (150 and 250 mg/kg) of EEAV and standard drug, respectively. The mean survival time (MST) and the effect of EEAV (150 and 250 mg/kg) at different doses on tumor volume, viable, and nonviable cell count, were shown in Tables 2 and 3 and graphical representation are shown in Figures 2 and 3.

**Table 2:** Effect of ethanol extract of *Adiantum venustum* on survival time on EAC-bearing mice

Experimental groups	Mean survival time (MST), days	% Increase in life span
Normal control GRP 1	-	-
EAC control GRP 2	22 ± 0.25	-
150 mg/kg GRP 3	24 ± 0.33	9.09
250 mg/kg GRP 4	29 ± 0.49	31.81
STD GRP 5	31 ± 0.55	40.90

Values are mean ± SEM. Number of mice in each group (*n* = 8), *P* < 0.001. Experimental group was compared with EAC control

**Table 3:** Effect of ethanol extract of *A. venustum* on tumor volume, packed cell volume, viable, and nonviable tumor cell count of EAC-bearing mice

Parameters	Normal GRP 1	EAC Control GRP 2	150 mg/kg GRP 3	250 mg/kg GRP 4	Standard GRP 5
Body weight	26.11 ± 0.12	26.11 ± 0.12	24.34 ± 0.16	23.28 ± 0.13	23.9 ± 0.02
Tumor volume (mL)	0	5.82 ± 0.042	4.22 ± 0.051	3.42 ± 0.082	2.42 ± 0.13
Packed cell volume (mL)	0	2.12 ± 0.104	1.75 ± 0.043	1.05 ± 0.092	1.15 ± 0.03
Viable tumor cell count, % 10^7^ cells /mL	0	11.25 ± 0.098	7.78 ± 0.18	4.85 ± 0.23	4.90 ± 0.015
Nonviable tumor cell count, ×10^7^ cells/mL	0	0.5 ± 0.017	0.92 ± 0.023	1.47 ± 0.021	1.23 ± 0.81

Values are mean ± SEM. Number of mice in each group (*n* = 8), *P* < 0.01. Experimental groups was compared to EAC control weight of normal mice = 20 ± 0.15

#### Effect EEAV on hematological parameter

**Figure 4:**
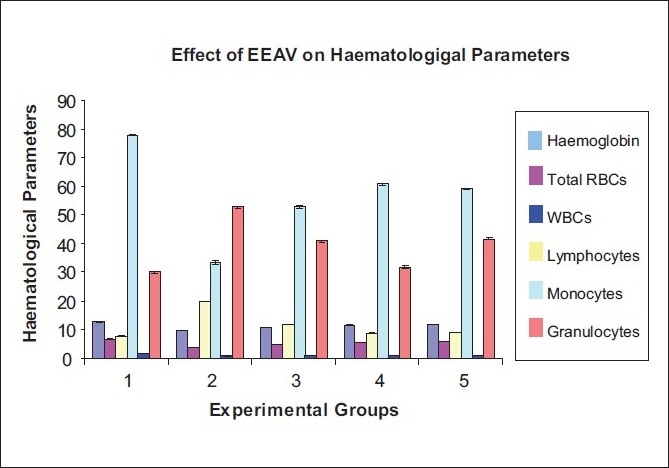
Graphical representation of effect of Ethanolic Extract of *A. venstrun* don on haematological parameters

EEAV at the dose of 100 and 200 mg/kg, the hemoglobin content in EAC-bearing mice were increased to 10.6 ± 0.057 and 11.45 ± 0.057. The hemoglobin content in the EAC control mice (9.8 ± 0.02) was significantly decreased as compared to normal mice (12.85 ±0.25). The total WBC count was significantly higher in the EAC-treated mice when compared to normal mice. Whereas EEAV-treated mice significantly reduced the WBC count as compared to that of control mice. Significant changes observed on differential count when extract-treated mice compared to EAC control mice [Table 4] [Figure 4].

**Table 4:** Effect of ethanol extract of *Adiantum venustum* on hematological parameters of EAC-treated mice

Parameter	Normal GRP 1	EAC control GRP 2	150 mg/kg GRP 3	250 mg/kg GRP 4	Standard GRP 5
Hemoglobin (g)	12.85 ± 0.25	9.8 ± 0.02	10.6 ± 0.057	11.45 ± 0.18*	11.7 ± 0.045*
Total RBC million/mmcu	6.65 ± 0.18	3.8 ± 0.035	4.75 ± 0.032	5.42 ± 0.22*	5.8 ± 0.054
Total WBC million/mmcu	7.8 ± 0.045	20.07 ± 0.068*	11.92 ± 0.042	8.85 ± 0.059	9.12 ± 0.055
Lymphocyte	77.75 ± 0.19	33.37 ± 0.56*	52.7 ± 0.50*	60.72 ± 0.36*	59.12 ± 0.30
Monocyte	1.7 ± 0.035	0.82 ± 0.024	1.15 ± 0.014*	1.2 ± 0.045	1.32 ± 0.024
Granulocyte	29.97 ± 0.46	52.6 ± 0.37*	40.87 ± 0.2	31.72 ± 0.63*	41.65 ± 0.29

Values are mean±SEM, (*n* = 8). EAC control group compared to normal group. Experimental group compared to EAC control. *P* < 0.05,

* *P* < 0.05

#### Biochemical assay

**Figure 5:**
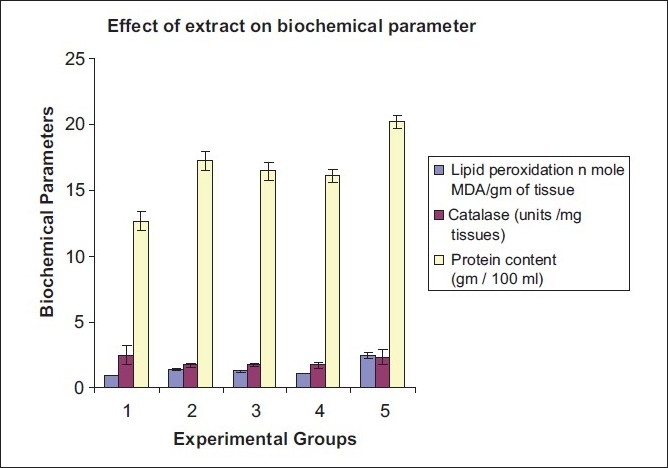
Graphical representation of effect of EEAV on different biochemical parameters

Biochemical assay indicated that EEAV significantly reduced the elevated levels of lipid peroxidation, and thereby it may act as an antitumor agent. The level of lipid peroxidation, catalase, and protein content were summarized in Table 5 and graphical representation is shown in Figure 5.

**Table 5:** Effect of different doses of ethanolic extract of *Adiantum venustum* on different biochemical parameter in EAC-bearing mice

Parameter	Normal GRP 1	EAC control GRP 2	150 mg/kg GRP 3	250 mg/kg GRP 4	Standard GRP 5
Lipid peroxidation *n* mole MDA/g of tissue	0.92±0.02	1.36±0.09*	1.27±0.04*	1.13±0.02	2.45±0.25
Catalase (units/mg tissues)	2.51±0.72	1.71±0.15*	1.75±0.13	2.34±0.23*	3.56±0.63
Protein content (g/100 mL)	12.66±0.69*	17.25±0.76	16.50±0.70	16.10±0.55	20.24±0.47

Values are mean±SEM, (*n* = 8). EAC control group compared to normal group. Experimental group compared to EAC control. *P* < 0.05,

* *P* < 0.05

## DISCUSSION

This study was carried out to evaluate the antitumor effect and antioxidant status of plant extract in EAC-bearing mice. The plant extract-treated animals at the doses of 150 and 250 mg/kg significantly inhibited the tumor volume, packed cell volume, tumor cell count, and brought back the hematological parameters to more or less normal levels. The extract also restored the hepatic lipid peroxidation and antioxidant enzymes such as CAT in tumor-bearing mice to near normal levels. In short-term toxicity studies, the administration of plant extract at the dose of 150 and 250 mg/kg for 14 days did not exhibit any adverse effect. In EAC-bearing mice, a regular rapid increase in ascites tumor volume was noted. Ascites fluid is the direct nutritional source for tumor cells and a rapid increase in ascitic fluid with tumor growth would be a means to meet the nutritional requirement of tumor cells.[21] Treatment with plant extract inhibited the tumor volume, tumor cell count, and increased the percentage of tryphan blue positive stained dead cells in tumor-bearing mice. The reliable criteria for judging the value of any anticancer drug are the rolongation of the life span of animals.[22] The plant extract decreased the ascites fluid volume, viable cell count, and increased the percentage of life span. It may be concluded that plant extract by decreasing the nutritional fluid volume and arresting the tumor growth increases the life span of EAC-bearing mice. Usually, in cancer chemotherapy the major problems that are being encountered are of myelosuppression and anemia.[23,24] The anemia encountered in tumor-bearing mice is mainly due to reduction in RBC or hemoglobin percentage, and this may occur either due to iron deficiency or due to hemolytic or myelopathic conditions.[25,26] Treatment with plant extract brought back the hemoglobin content, RBC, and WBC count more or less to normal levels. This indicates that plant extract possesses protective action on the hemopoietic system. Lipid peroxidation, an autocatalytic free-radical chain propagating reaction, is known to be associated with pathological conditions of a cell. Malondialdehyde (MDA), the end product of lipid peroxidation, was reported to be higher in cancer tissues than in nondiseased organ.[27] Excessive production of free radicals resulted in oxidative stress, which leads to damage of macromolecules, for example lipid peroxidation *in vivo*.[28] It was also reported that the presence of tumors in the human body or in experimental animals is known to affect many functions of the vital organs, especially in the liver, even when the site of the tumor does not interfere directly with organ function.[29] Plant extract significantly reduced the elevated levels of lipid peroxidation and increased the glutathione content in EAC-treated mice. The antitumerogenic effect of plant extract may be due to the antioxidant, and the free-radical quenching property of the phytoconstituents of plant extract. Cells are also equipped with enzymatic antioxidant mechanisms that play an important role in the elimination of free radicals. SOD, CAT, and glutathione peroxides are involved in the clearance of superoxide and hydrogen peroxide (H_2_ O_2_). SOD catalyses the diminution of superoxide into H_2_ O_2_, which has to be eliminated by glutathione peroxidase and/or catalase.[30] A small amount of catalase in tumor cells was reported.[31] The inhibition of SOD and CAT activities as a result of tumor growth were also reported.[32] Similar findings were observed in this study with EAC-bearing mice. The administration of plant extract at different doses significantly increased CAT levels in a dose-dependent manner. It was reported that plant-derived extracts containing antioxidant principles showed cytotoxicity toward tumor cells[33] and antitumor activity in experimental animals.[34] Antitumor activity of these antioxidants is either through induction of apoptosis[35] or by inhibition of neovascularization.[36] The implication of free radicals in tumors is well documented.[37] The free-radical hypothesis supported the fact that the antioxidants effectively inhibit the tumor, and the observed properties may be attributed to the antioxidant and antitumor principles present in the extract. This study demonstrates that plant extract increased the life span of EAC-tumor-bearing mice and decreased the lipid peroxidation and thereby augmented the endogenous antioxidant enzymes in the liver. The above-mentioned parameters are responsible for the antitumor and antioxidant activities of *A. venstrum don*. Further investigations are in progress in our laboratory to identify the active principles involved in this anticancer and antioxidant activity.

## CONCLUSION

The EEAV possessed significant anticancer and antioxidant activity due to the presence of terpenoids and flavonoids. Further investigation on various biological activities of this plant with different modes will not only validate the types of activities claimed by Ayurvedic, Siddha, and traditional practitioners, but also will bring out innovation in the field of therapeutics.

## References

[1] Kadam SS, Mahadik KR, Bothra KG (1989). “Principle of Medicinal Chemistry”, Nirali prakashan.

[2] Kirtikar KP, Basu BD (1935). Indian Medicinal plants.

[3] Natkarni KM (1976). Indian Materia Medica.

[4] Ambarta SP (1986). The Useful plants of India.

[5] Harbone JB (2005). Phytochemical Method, A Guide to modern techniques of plant Analysis.

[6] Krishnaswamy NR (2003). Chemistry of Natural products, A laboratory hand book.

[7] Mohammad A (2000-2001). Objective type pharmacy.

[8] Khandelwal KR, Kokate CK, Pawar AP, Gokhle SB (1996). Practical pharmacognosy Techniques and Experiments.

[9] Rodney RW, Thomas AB, Donald VL (1992). Antimicrobial testing, toxicity testing and safety determination for twelve antimicrobials with penaeid shrimp larvae. J Aquat Anim Health.

[10] Margarita FT, Ángeles SP, Dolores FM (2004). Acute ld_50_ of a *gyrodinium corsicum* natural population for *sparus aurata* and *dicentrarchus labrax*. Harmful Algae.

[11] Jung H, Choi Sc (1994). Sequential method of estimating the LD_50_ using a modified up-and-down rule. J Biopharm Stat.

[12] Teresa K, Joseph S (1995). Preparative Layer Chromatography, chromatographic science series.

[13] Ng TB, Gao W, Li L, Niu SM, Zhao L, Liu J (2005). Rose (*Rosa rugosa*) - flower extract increases the activities of antioxidant enzymes and their gene expression and reduce lipid peroxidation. Biochem Cell Biol.

[14] Nicol BM, Prasad SB (2006). The effect of cyclophosphamide alone and in combination with ascorbic acid against murine ascites Dalton’s lymphoma. Indian J Pharmacol.

[15] Horvathova K, Novotny L, Tothova D, Vachalkova A (2004). Determination of free radical scavenging activity of quercetin, rutin, luteolin and apigenin in H2O2-treated human ML cells K562. Neoplasma.

[16] Kavitha K, Manoharan S (2006). Anticarcinogenic and antilipidperoxidative effect of *Tephrosia purpurea* (Linn). Pers, in 7,12- dimethyl benz (a) anthracene (DMBA) induced hamster buccal pouch carcinoma. Indian J Pharmacol.

[17] Duh PD, Tu YY, Yen GC (1999). Antioxidant activity of water extracts of Harng Jyur (*Chrisanthemun morifolium Ramat*). Lebensm Wiss Technol.

[18] Khanam JA, Bag SP, Sur B, Sur P (1997). Antineoplastic activity of copper benzohydroxamic and complex against Ehrlich ascites carcinoma (EAC) in mice. Indian J Pharm.

[19] Neves M, Morais R, Gafner S, Hostettmann K (1998). Three triterpenoids and one flavonoid from the liverwort *Asterella blumeana* grown *in vitro*. Phytother Res.

[20] Kumarappan CT, Mandal SC (2007). Antitumor activity of polyphenolic extract of *ichnocarpus frutescens*. Exp Oncol.

[21] Prasad SB, Giri A (1994). Antitumor effect of cisplatin against murineascites Dalton’s lymphoma. Indian J Expt Biol.

[22] Clarkson BD, Burchenal JH (1965). Preliminary screening of antineoplastic drugs. Prog Clin Cancer.

[23] Price VE, Greenfield RE (1958). Anemia in cancer. Adv Cancer Res.

[24] Hogland HC (1982). Hematological complications of cancer chemotherapy. Semin Oncol.

[25] Fenninger LD, Mider GB (1954). Energy and nitrogen metabolism in cancer. Adv Cancer Res.

[26] Yagi K (1987). Lipid peroxides and human diseases. Chem Phys Lipids.

[27] Sinclair AJ, Barnett AH, Lunie J (1990). Free radical and auto-oxidant systems in health and disease. Br J Hosp Med.

[28] DeWys WD (1982). Pathophysiology of cancer cachexia: current understanding and areas for future research. Cancer Res.

[29] Rushmore TH, Picket CB (1993). Glutathione-S-transferase, structure, regulation, and therapeutic implication. J Biol Chem.

[30] Sun Y, Oberley LW, Elwell JH, Sierra Rivera E (1989). Antioxidant enzyme activities in normal and transformed mice liver cells. Int J Cancer.

[31] Marklund SL, Westman NG, Lundgren E, Roos G (1982). Copper- and zinc-containing superoxide dismutase, catalase, and glutathione peroxidase in normal and neoplastic human cell lines and normal human tissues. Cancer Res.

[32] Jiau-Jian L, Larry WO (1977). Over expression of manganese-containing superoxide dismutase confers resistance to the cytotoxicity of tumor necrosis factor _ and/or hyperthermia. Cancer Res.

[33] Ruby AJ, Kuttan G, Babu KD, Rajasekaran KN, Kuttan R (1995). Antitumor and antioxidant activity of natural curcuminoids. Cancer Lett.

[34] Ming L, Jill CP, Jingfang JN, Edward C, Brash E (1998). Antioxidant action via p53 mediated apoptosis. Cancer Res.

[35] Putul M, Sunit C, Pritha B (2000). Neovascularisation offers a new perspective to glutamine-related therapy. Indian J Exp Biol.

[36] Ravid A, Korean R (2003). The role of reactive oxygen species in the anticancer activity of vitamin D. Anticancer Res.

[37] Feng Q, Kumangai T, Torii Y, Nakamura Y, Osawa T, Uchida K (2001). Anticarcinigenic antioxidants as inhibitors against intracellular oxidative stree. Free Radic Res.

